# Oxford Nanopore Sequencing, a Promising Technology for Precision Diagnostics in Intensive Care Units: A Narrative Review

**DOI:** 10.3390/biomedicines14091910

**Published:** 2026-08-26

**Authors:** Leonard Azamfirei, Dorin Bica, Maier Alexandru Mihai, Balla Beata, Claudia Banescu

**Affiliations:** 1 Department of Anaesthesiology and Intensive Care Medicine, George Emil Palade University of Medicine, Pharmacy, Science, and Technology of Targu Mures, Gheorghe Marinescu 38, 540139 Targu Mures, Romania; leonard.azamfirei@umfst.ro; 2 Department of Electrical Engineering and Information Technology, George Emil Palade University of Medicine, Pharmacy, Science, and Technology of Targu Mures, Gheorghe Marinescu 38, 540139 Targu Mures, Romania; 3 Center for Advanced Medical and Pharmaceutical Research, George Emil Palade University of Medicine, Pharmacy, Science, and Technology of Targu Mures, 540139 Targu Mures, Romania; maier.mihai.a@gmail.com (M.A.M.); beata.balla@umfst.ro (B.B.); claudia.banescu@umfst.ro (C.B.)

**Keywords:** nanopore technology, sequencing, intensive care unit

## Abstract

**Background**: Precision diagnostics are more and more important in intensive care units (ICUs), where rapid identification of infectious agents and antimicrobial resistance determinants is crucial for timely and appropriate treatment. Conventional microbiological methods are frequently limited by long turnaround times and reduced sensitivity, which may delay appropriate treatment. Nanopore sequencing allows rapid, direct, and long-read sequencing of DNA/RNA molecules without the need for amplification, avoiding biases introduced by NGS during amplification and library preparation and generating data in real time. **Objectives**: This narrative review aims to summarize current knowledge of nanopore technology in the ICU, discuss nanopore principles and current clinical applications in intensive care medicine, highlight its advantages and limitations, and explore future perspectives for integrating nanopore-based diagnostics into precision critical care. **Methods**: A literature search was performed using PubMed and Web of Science. The literature search was conducted with no lower restriction, covering English-language publications. **Results**: Nanopore sequencing enables real-time, long-read, single-molecule analysis of native nucleic acid molecules, rapid pathogen identification, antimicrobial resistance profiling, metagenomic analysis, and direct sequencing without amplification. Recent studies have proved the clinical utility of nanopore sequencing in critically ill patients with sepsis, bloodstream infections, hospital-acquired pneumonia, ventilator-associated pneumonia, and fungal and viral infections. Its portability, rapid turnaround time, and potential for point-of-care implementation make it particularly attractive for ICU settings. **Conclusions**: Nanopore sequencing technology represents a promising molecular diagnostic tool, but wider clinical implementation warrants further larger studies with clinical outcome endpoints, standardized bioinformatic pipelines, and clearer validation pathways.

## 1. Introduction

Patients admitted to intensive care units (ICUs) are always critically ill and highly susceptible to different infections, such as bloodstream infections, catheter-associated urinary tract infections, and ventilator-associated pneumonia [1], or are diagnosed with sepsis.

Sepsis is a major cause of morbidity and mortality due to organ dysfunction caused by a dysregulated host response to infection [2,3]. To benefit from appropriate treatment and to ensure favorable outcomes, rapid pathogen identification and antimicrobial susceptibility phenotyping are mandatory, particularly in the increasing prevalence of antimicrobial resistance [4]. Antimicrobial resistance (AMR) further compounds the burden of ICU-associated infections. Recent Global Burden of Disease estimates attribute more than one million deaths annually to bacterial AMR, with projections exceeding 39 million by 2050 [5]. This threat is most pronounced in critical care, where a recent Italian study reported carbapenemase resistance of 55.0% in Klebsiella pneumoniae and multidrug resistance of 97% in Acinetobacter baumannii, both associated with higher bloodstream-infection mortality [6], directly narrowing empirical options and worsening sepsis outcomes.

Considering that the available pathogen identification and culture-based antimicrobial susceptibility testing can take about 3–5 days and also that no pathogen is isolated in almost 30% of sepsis episodes and around two-thirds of community-acquired pneumonia, rapid molecular detection and gene profiling technologies are needed [4,7]. Likewise, conventional biomarker-based methods used in ICU and emergency departments [8] are limited by factors such as a risk of false-positive results, high costs, and availability, which emphasizes the need for a complementary fast molecular diagnosis approach.

Therefore, survival in the intensive care unit (ICU) is directly dependent on the rapidity of initiating the appropriate antimicrobial therapy. Every hour of treatment delay increases mortality [9]. This results in an urgent, unmet clinical need for appropriate, novel, and effective technologies capable of identifying infectious agents and their resistance profiles within a few hours.

Next-generation sequencing (NGS) technologies allow direct analysis of microbial nucleic acids, detecting pathogens independent of culture growth and those that might otherwise remain unidentified by conventional methods [10]. Several sequencing strategies have been proposed for diagnostics in critically ill patients in the ICU, including targeted next-generation sequencing (tNGS), whole-genome sequencing (WGS), and metagenomic next-generation sequencing (mNGS) [10,11,12].

Among the available sequencing technologies, Oxford Nanopore Technologies has attracted particular attention because of its unique technical characteristics. Nanopore sequencing technology allows rapid and long-read sequencing, generates data in real time, supports portable devices, allows direct sequencing of DNA or RNA molecules without the need for extensive amplification steps and avoids biases introduced by NGS during amplification and library preparation [13]. The characteristics of nanopore technologies are critical in the ICU, where rapid turnaround time (TAT) and bedside applicability are key aspects.

The available data underlines the clinical utility of nanopore technology for pathogen identification, detection of antimicrobial resistance, microbiome analysis, characterization of host responses and enabling tissue-of-origin and pathogen detection in plasma cell-free DNA from critically ill patients [13,14,15,16].

Do we need rapid sequencing in the ICU?

Nanopore sequencing technologies provide not only fast pathogen identification but may also provide relevant information on antimicrobial resistance (AMR) determinants, such as genes associated with carbapenem resistance [17].

This may allow earlier personalized antimicrobial treatment and strengthen antimicrobial stewardship initiatives [18].

Nanopore technology represents a fast, cost-effective, and portable solution for bacterial genome sequencing, particularly valuable when rapid genetic diagnosis is required [19].

The principal advantage of Oxford Nanopore Technology (ONT) sequencing is its ability to generate clinically actionable information within hours rather than days. Unlike conventional culture-based diagnostics, nanopore sequencing enables simultaneous pathogen identification and antimicrobial resistance gene detection directly from clinical specimens. This shorter turnaround time could enable earlier optimization of antimicrobial therapy and improve antimicrobial stewardship in critically ill patients [20].

Critically ill patients frequently associate multiple comorbidities [21], nonspecific clinical manifestations before antimicrobial treatment, and an increased prevalence of multidrug-resistant pathogens. In this situation, establishing a diagnosis remains difficult despite advances in conventional laboratory testing, and undertreatment of resistant pathogens may be observed. These findings have generated growing interest in the integration of sequencing technologies into routine critical care practice. Studies performed by Alcolea-Medina et al. [15] and Bay et al. [7] have shown that sequencing-based diagnostics can improve pathogen detection and may influence therapeutic decision-making.

Nevertheless, both studies have important design limitations: the study conducted by Alcolea-Medina was single-center and observational, while the study performed by Bay et al. was multicenter but retrospective; thus, their observations on therapeutic impact need further validation through prospective, where possible randomized or controlled, multicenter study designs [7,15].

Therefore, rapid molecular detection and resistance gene profiling methodologies are needed to guide clinical management, improve prognosis, optimize antimicrobial treatment and reduce time to pathogen identification and costs in critically ill patients, especially in the era of an increasing prevalence of antimicrobial resistance (AMR) [4]. AMR has significant cost implications for healthcare systems worldwide, primarily due to increased costs of managing resistant infections. Earlier administration of appropriate antimicrobial therapy has been associated with improved outcomes in critically ill patients. Recent prospective studies conducted in ICU cases have demonstrated increasing clinical utility of sequencing approaches [20,22].

Healthcare-associated infections remain one of the leading causes of morbidity and mortality among critically ill patients. The increasing prevalence of multidrug-resistant organisms, combined with the limited sensitivity and prolonged turnaround time of conventional microbiological methods, frequently delays appropriate antimicrobial therapy. These diagnostic limitations have driven the development of rapid sequencing-based approaches capable of identifying pathogens and antimicrobial resistance determinants within clinically actionable timeframes [18,20].

This narrative review aims to summarize current knowledge of nanopore sequencing technology in intensive care units, focusing on the technical principles of the platform, library preparation and sequencing workflow, bioinformatic analysis pipeline, available sequencing platforms, clinical applications in the ICU, limitations, and perspectives for integration of nanopore-based diagnostics into precision critical care.

## 2. Materials and Methods

This narrative literature review synthesizes the current evidence regarding the role of nanopore sequencing technologies in intensive care units. This review explores the role of nanopore technology for rapid diagnostic sequencing in supporting clinical decision-making in critical care patients. A literature search was performed using the following databases: PubMed and Web of Science. The literature search was conducted with no lower restriction, covering publications up to June 2026, using the following keywords: “nanopore”, “sequencing”, “Oxford Nanopore”, “third-generation sequencing”, “intensive care”, “critically ill”, and “ICU”. Inclusion criteria were: articles published in English, original research articles, systematic reviews, narrative reviews, editorials, and protocols. Exclusion criteria were: non-English articles, conference abstracts, duplicate publications, and studies not directly related to nanopore sequencing and the ICU. Approximately 140 publications were initially identified. After duplicate removal and title/abstract screening, 87 articles were considered relevant and included in the narrative synthesis. Studies describing clinical applications of nanopore sequencing in critically ill patients, intensive care medicine, pathogen identification, antimicrobial resistance detection, or related methodological advances were prioritized. Additional relevant publications were identified through manual screening of reference lists. As this was a narrative review, no formal PRISMA workflow or quantitative quality assessment was performed.

## 3. Nanopore Sequencing Technology

### 3.1. Nanopore Principles

Nanopore single-molecule sequencing (SMS) is a third-generation technology that relies on electrophoretically driven translocation of DNA/RNA molecules through biologically engineered nanopore protein channels embedded within an electroresistant synthetic membrane [23]. As each molecule passes through the pore, it perturbs the ionic current in a characteristic way, generating a measurable electrical signal that allows label-free, real-time molecular identification [23]. Beyond nucleic acid sequencing, the nanopore technology may be used to characterize a broad range of other molecules and molecular complexes, and its combination of high throughput, high resolution, reproducibility, low cost, and strong technical compatibility allows target genes and disease biomarkers to be identified in shorter timeframes than conventional methods, therefore supporting faster, more accurate clinical diagnosis [24].

In order to understand the sequencing process, it is important to highlight each of the five interconnected components and their workflow from an engineering perspective. The components can be divided into: the membrane (the platform on which the sequencing process occurs), the nanopore (the sensor), the motor protein (the molecular speed controller), the applied voltage (the driving force), and the signal processing and basecalling (the decoding system of the platform).

The membrane is a synthetic polymer and acts as an electrical insulator between two chambers filled with electrolyte solution. The nanopore, located at the membrane level, is the only connecting gate between the solutions and is the sensing part of the platform. The motor protein sits atop of the nanopore and has the role of regulating the transportation speed (which is dictated by the applied voltage) through the nanopore at a frequency that can be electrically sampled. The result of the electrically sampled signal is called the squiggle; its translation is the basecalling and the signal processing part, offering the final output.

Double-stranded DNA (dsDNA) is unwound by a motor protein (that has helicase activity) before translocation. Application of a constant voltage (usually ~180 mV) across the membrane drives single-stranded DNA (ssDNA) or RNA through nanoscale pores (with different pore sizes and surface properties that naturally form channels that may be used for sequencing). Under these conditions, ions flow continuously across the nanopore (from the negatively charged “cis” side to the positively charged “trans” side), producing a stable baseline current. As DNA molecules move through the pore, they partially obstruct ion flow, generating characteristic changes in the ionic current that correspond to the sequence composition of the translocating DNA strand [25].

**Figure 1:**
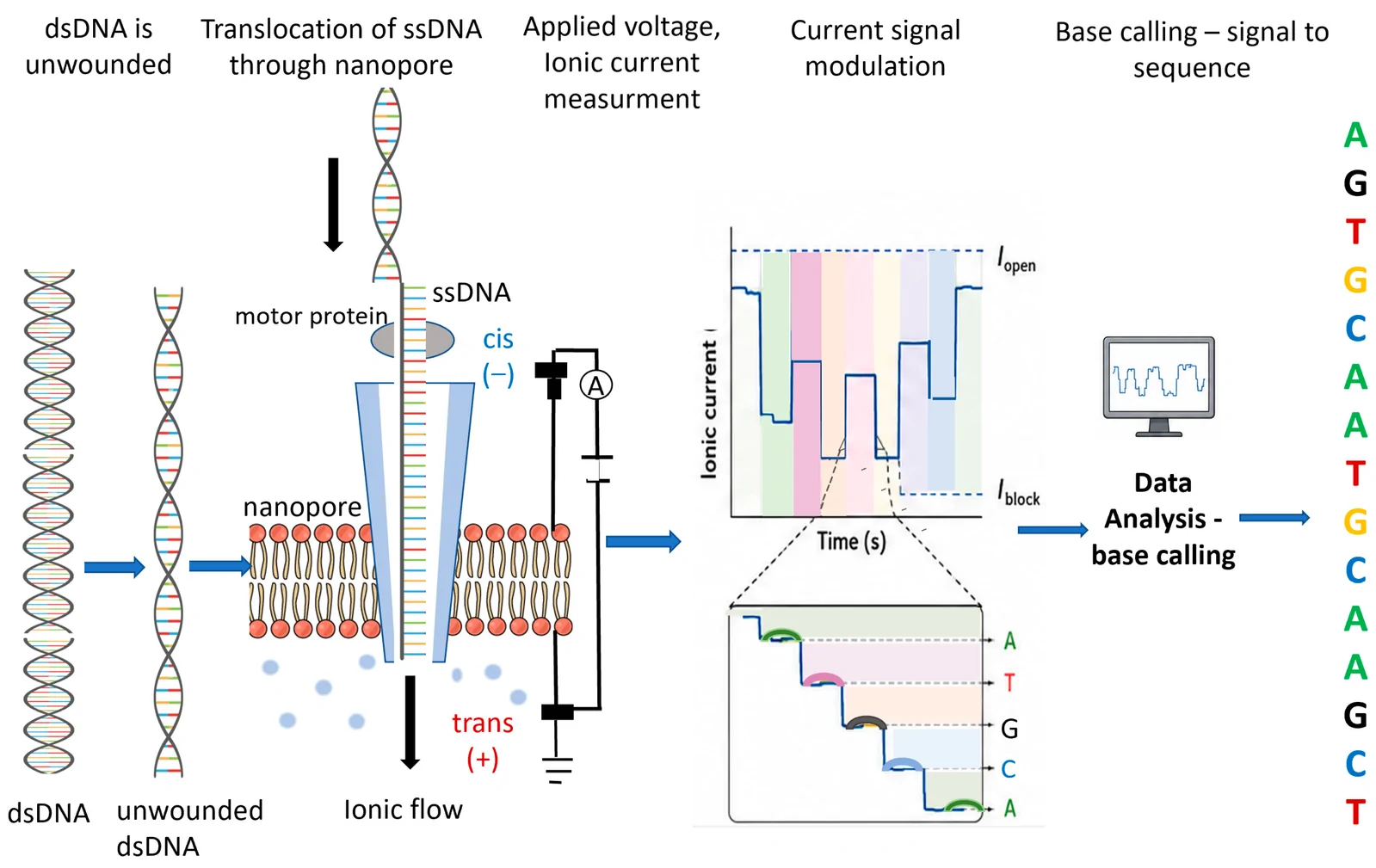
Schematic overview of nanopore sequencing. dsDNA is unwound by a motor protein, which acts as a helicase, and ssDNA is translocated through a nanopore after applying a constant voltage. Changes in ionic current produced by each nucleotide are recorded and converted into nucleotide sequences by basecalling software (dsDNA—double-stranded DNA, ssDNA—single-stranded DNA, A—Adenine, G—Guanine, T—Thymine, C—Cytosine) (https://nanoporetech.com/software/categories/standalone-basecalling-software, accessed on 4 July 2026).

Each nucleotide traverses the channel’s narrowest region in a linear sequence and induces a transient, base-specific disruption in the ionic current, generating characteristic electrical signals (unique patterns, commonly termed as a “squiggle”), which are captured in real time and interpreted by machine learning algorithms to reconstruct the nucleotide sequence. A schematic workflow of nanopore sequencing is presented in Figure 1.

The sequencing process begins with applying an electrical potential across the membrane containing the protein nanopores. The resulting ionic current flows through the nanopore, and therefore, the primary measurable base signal is obtained. Because nucleic acids possess a negatively charged phosphate backbone, the induced electric field also drives DNA or RNA molecules through the pore. As nucleotides pass through the sensing region, they partially disrupt ion flow and generate current changes that reflect the underlying nucleotide sequence.

The incorporation of motor proteins at the nanopore level regulates nucleic acid translocation through the pore, enabling nanopore sequencing [26]. Without these proteins, DNA traverses nanopores too rapidly for accurate signal acquisition. They slow and control strand movement, allowing electronic systems to record sequence-dependent current fluctuations with sufficient resolution for data analysis [27]. This controlled translocation is essential for achieving accurate sequence determination.

As already established, the performance of nanopore sequencing is directly influenced by the architecture of the pore itself. Nanopores can be classified into two categories: biological- and solid-state nanopores. Biological nanopores are transmembrane protein channels, and the most extensively characterized are the heptameric α-hemolysin from Staphylococcus aureus (the first and most widely used biological nanopore, but its relatively long sensing region reduces single-base resolution), the octameric MspA porin from Mycobacterium smegmatis (which has a much shorter and narrower constriction and is the preferred pore for high-resolution applications), and the dodecameric connector channel from bacteriophage phi29 [28].

The pore chemistry applied by ONT has evolved substantially. Current commercial flow cells are based on engineered CsgG nanopores (derived from Escherichia coli CsgG), whose well-defined constriction at the center modulates the ionic current as molecules pass through. The R9 chemistry, based on engineered CsgG, achieved accurate identification of homopolymers up to five nucleotides in length; a subsequent dual-constriction pore combining CsgG with its interaction partner CsgF extended accurate homopolymer resolution to nine nucleotides and improved basecalling accuracy by 25–70% [24]. The most recent R10.4.1 chemistry, featuring a longer dual-reader head, has further improved discrimination in homopolymeric and repetitive regions, which historically accounted for a large proportion of sequencing errors [24]. In practice, the choice of flow-cell chemistry directly determines raw read accuracy, homopolymer handling, and the reliability of downstream AMR calling.

The primary output of the system is a current-versus-time trace, referred to as a “squiggle”. Rather than representing individual nucleotides directly, each signal measurement reflects the combined influence of several neighboring bases occupying the sensing nanopore region simultaneously [29]. Consequently, nanopore sequencing generates complex electrical patterns that must be translated into nucleotide sequences with the help of computational power. This translation process, known as basecalling, has evolved substantially in recent years with major advances in deep learning. Current nanopore platforms employ algorithms trained to recognize relationships between electrical signals and nucleotide sequences [30]. Machine learning-based basecalling has enhanced sequencing accuracy and contributed to the increasing clinical applicability of nanopore sequencing for pathogen identification, antimicrobial resistance detection, and genomic characterization.

From a critical care perspective, the most important consequence of these engineering innovations is the ability to generate clinically relevant information in real time. Unlike conventional sequencing workflows that typically require completing an entire sequencing run before analysis can begin, nanopore sequencing produces interpretable data continuously as molecules are being sequenced, making nanopore sequencing particularly attractive for intensive care environments where timely therapeutic decisions are critical [31].

### 3.2. Library Preparation

It is characterized by a versatile workflow consisting of fewer processing steps than most short-read sequencing platforms, reducing both laboratory workload and sample processing time. Libraries may be generated within minutes, supporting rapid pathogen identification in critically ill patients by enabling PCR-free direct DNA or RNA sequencing.

Library preparation ensures the sequencing-compatible format of the nucleic acids by attaching them to the motor protein (which associates with the nanopore and controls nucleic acids) and corresponding adapters (oligonucleotides). It involves ligation-based library preparation (using the Ligation Sequencing Kit to attach the adapter), which preserves long DNA fragments, making it suitable for antimicrobial resistance characterization and identification of native base modifications [32]. It consists of a PCR-free protocol and requires ≥100 ng of nucleic acid.

Rapid library preparation (using the Rapid Barcoding Kit) combines DNA fragmentation and adapter attachment in a single step, thereby shortening processing time and making it highly reliable for ICU diagnostics [32]. PCR amplification is required when there is < 100 ng of nucleic acid, but this may lead to PCR bias or polymerase error and not preserve native base modifications.

For multiple samples sequenced in a single run, barcoding is necessary. Barcoding enables multiple samples (multiplexing) to be pooled and sequenced simultaneously on a single flow cell, with reads subsequently assigned to individual samples through barcode-based demultiplexing. This enables the re-identification of sequencing data specific to each sample for further analysis [33] and earlier pathogen identification in ICU workflows.

For library preparation, Rapid Barcoding Sequencing or an appropriate Nanopore Genomic Ligation kit with Native barcoding is used, and the resulting libraries are loaded onto the flow cell and run on the device.

Nucleic acid quality and quantity requirements are as follows: for optimal nanopore sequencing results, high-quality, high-molecular-weight DNA with ≥100 ng is recommended, although rapid library preparation kits can accommodate lower DNA inputs. For RNA pathogens, RNA is usually transcribed into complementary DNA (cDNA) before sequencing, providing higher yields and more reliable results than direct RNA sequencing.

### 3.3. Bioinformatics Analysis

The bioinformatics analysis of data obtained through nanopore sequencing involves more steps that ensure the conversion of raw electrical signals into results (base sequences).

#### 3.3.1. Signal Acquisition

Raw data generated as DNA or RNA strands pass through the nanopore are recorded (“squiggles”) and stored in POD5 (previously FAST5) files, with each file corresponding to a single sequencing read [34]. Data acquisition is performed by MinKNOW software version 26.01.15.

#### 3.3.2. Basecalling

Basecalling refers to the computational process by which the electrical current signals generated during nanopore sequencing are translated into nucleotide sequences (A, C, T, G). During DNA/RNA sequencing, the electrical signal changes are decoded using basecalling algorithms (for example, Guppy, Dorado) to determine the DNA or RNA sequence in real time [35].

Modern software solutions, such as Dorado version 2.1.1 (which uses deep learning algorithms), have substantially improved sequencing accuracy and can simultaneously identify modified bases, expanding the clinical and research applications of Oxford Nanopore Technology. It can run live during sequencing on GridION/PromethION or offline on GPU infrastructure [32]. Improvements in flow-cell chemistry (R10.4.1) and Dorado have increased raw read accuracy to over 99% [36].

To achieve competitive accuracies, basecalling requires neural networks [37]; therefore, these methods have improved significantly in recent years. Previous tools relied on hidden Markov models or recurrent neural networks combined with signal segmentation, which often led to errors [38], but modern approaches are end-to-end and avoid pre-segmentation. Deep learning (DL) approaches now decode the raw signal directly and achieve higher accuracy [39]. State-of-the-art basecallers, such as Guppy and Dorado (from Oxford Nanopore Technologies), are sequence context-free and can basecall a wide range of molecules [39]. Wick et al. consider that the choice of architecture and training data also plays an important role [30], and recent methods use incremental learning to correctly basecall sequences rich in modified bases [39].

Gao et al. reported that AI approaches ranging from traditional ML to modern DL have improved signal-to-noise ratio, interpretation of data and classification accuracy [40].

#### 3.3.3. Demultiplexing

By demultiplexing, each read is assigned to its sample of origin based on the attached barcode sequence, being performed by Dorado or the MinKNOW demultiplexer [32], producing individual FASTQ files for each sample and depending on a barcode score threshold (below which reads are classified as unassigned).

#### 3.3.4. Adapter Trimming and Quality Filtering

Residual sequencing adapters and barcodes are trimmed from the read ends before downstream analysis using tools such as Porechop (integrated trimming), and reads are filtered based on a minimum quality score. Low-quality reads are excluded using Chopper or similar software. It reduces sequencing artifacts and improves the accuracy of taxonomic classification and antimicrobial resistance detection [41]. In practice, reads are retained at a defined minimum quality (Q) threshold, for example, Q ≥ 9 in a recent metagenomic workflow [42], and short reads are discarded before downstream analysis (for example fragments ≤ 250 bp) [43].

#### 3.3.5. Host Read Depletion

Clinical samples frequently contain an increased proportion of host human DNA, sequencing reads are aligned against the human reference genome (GRCh38), and host-derived reads are removed, commonly using Minimap2. This increases the proportion of microbial reads [44]. Alignment is increasingly performed against the more complete telomere-to-telomere human reference (T2T-CHM13v2) rather than GRCh38 [42,43], and dedicated decontamination tools such as Hostile can further remove residual host reads while protecting patient privacy [45].

#### 3.3.6. Taxonomic Classification and Database Alignment

The microbial reads remaining after host read depletion are assigned to taxonomic classifications by comparing them with curated reference databases using tools such as Kraken2 (version 2.17.1), Centrifuge (version 1.0.4.2), Minimap2 (version 2.31), or the EPI2ME Desktop version 5.4.0 [46]. Taxonomic classification enables rapid identification of pathogens (bacteria, fungi, viruses, and parasites) directly from clinical specimens without prior culture.

To minimize contamination, negative sequencing controls are processed alongside clinical samples, and only microbial species whose abundance exceeds a predefined fold-change threshold relative to the negative control are retained. This approach is particularly important for low-biomass specimens, where contaminating reads originating from reagents or barcode cross-contamination may account for a substantial proportion of the sequencing output [42,44]. Furthermore, reliable taxonomic assignment is strengthened by applying predefined minimum read-count and relative-abundance thresholds before reporting a pathogen [43].

#### 3.3.7. Antimicrobial Resistance Gene Detection

After pathogen identification, the microbial reads are aligned against curated AMR databases, such as the Comprehensive Antibiotic Resistance Database or ResFinder, using tools such as AMRFinderPlus version 4.2.7, enabling detection of resistance and providing useful information to support antimicrobial stewardship. To minimize false positive results, an AMR determinant is typically reported only when it meets strict identity and coverage criteria (commonly ≥95–98%) and a minimum depth of coverage, since low-coverage regions can cause closely related genes to be misidentified [43].

Data analysis is commonly performed on an open integrated bioinformatics platform (EPI2ME from Oxford Nanopore Technologies). EPI2ME provides preconfigured, validated workflows for a variety of nanopore applications (including pathogen identification, metagenomic analysis and antimicrobial resistance detection).

**Figure 2:**
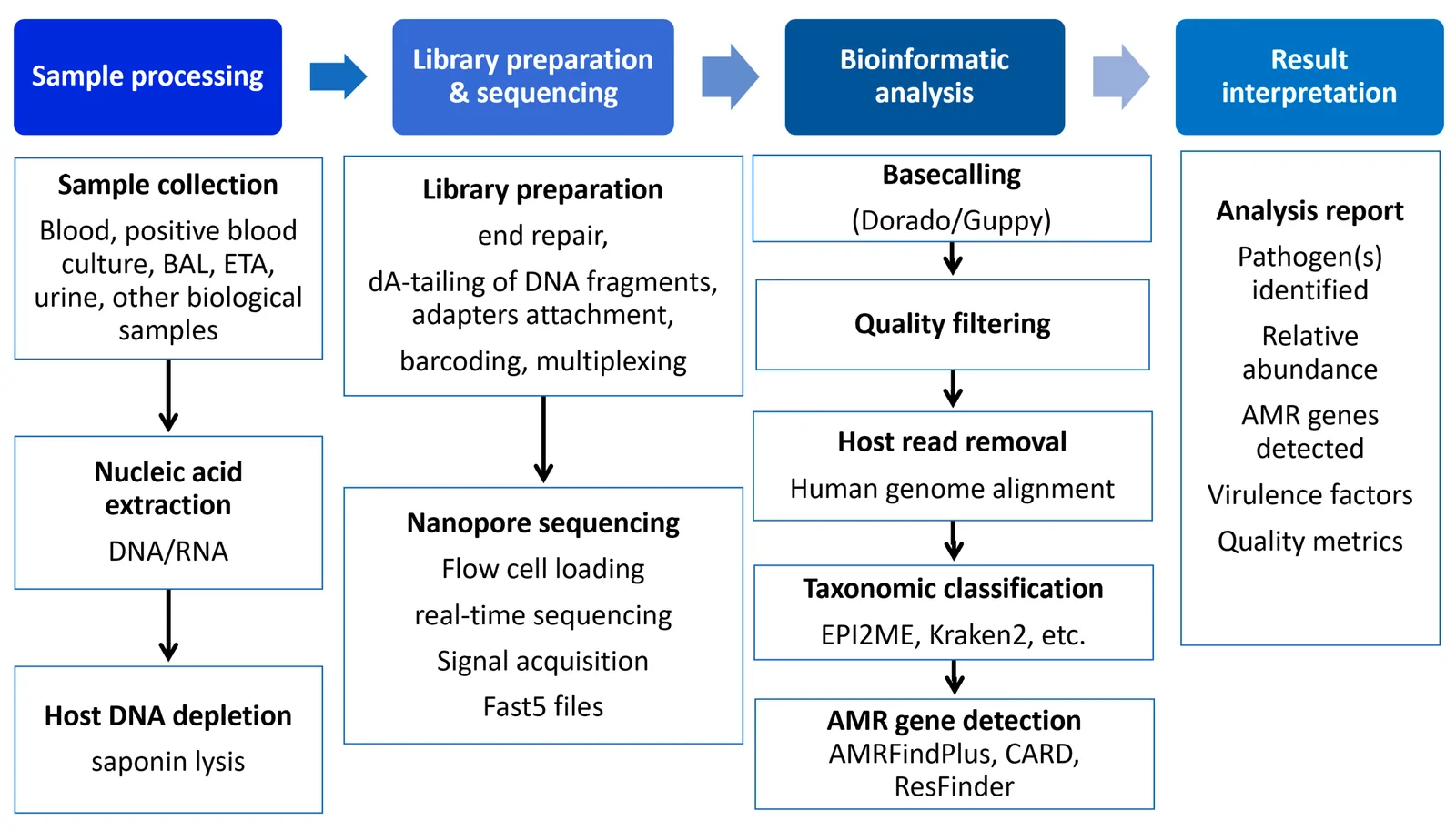
Workflow for rapid pathogen identification by nanopore sequencing—from sample collection to analysis report in ICU (BAL—bronchoalveolar lavage, ETA—endotracheal aspirate, AMR—antimicrobial resistance).

The complete analytical workflow for rapid pathogen identification by nanopore sequencing from clinical sample collection through library preparation, nanopore sequencing, bioinformatic processing, and final diagnostic reporting is summarized in Figure 2.

Although nanopore sequencing enables rapid identification of antimicrobial resistance genes, genotypic findings should not automatically be interpreted as phenotypic resistance. Gene expression, regulatory mechanisms, gene dosage, and previously undescribed resistance mechanisms may influence the observed phenotype. Consequently, discordant genotype–phenotype results should be interpreted cautiously and, whenever possible, confirmed by conventional antimicrobial susceptibility testing.

### 3.4. Nanopore Sequencing Platforms

There is a family of sequencing devices that use nanopore chemistry but differ substantially in throughput, scalability, computational capacity, and intended applications. The currently available platforms include MinION, GridION, and PromethION (Oxford Nanopore Technologies, Oxford, UK) [32,47].

MinION is the smallest instrument (pocket-sized, ~100 g), one of the most portable sequencing systems currently available, and the most widely used nanopore sequencing platform [47]. It is necessary to be connected to a computer via USB, has relatively low infrastructure requirements, and supports one flow cell. It allows sequencing in the lab or field near-patient without amplification and sequences any length of fragments, from short to ultra-long [48]. It is used for small genomes and targeted sequencing; pathogen identification may be achieved within hours of sequencing initiation [22]. It generates 10–15 gigabases (Gb), up to 50 Gb of data per run. It is suitable for rapid and near-patient diagnostics, including routine ICU applications [25,48].

Its portability and rapid turnaround time make the MinION uniquely suited to bedside or ICU, enabling point-of-care and bedside diagnostic testing.

GridION is a benchtop instrument for hospital laboratories or facilities that require higher throughput. It is recommended to be used for medium-scale projects, accommodating up to five independent flow cells (that can be used individually or together), generating up to 250 Gb of data per run (50 Gb/flow cell), and, with an integrated computer, supports real-time basecalling and bioinformatic analysis. It may be used by multiple users and different projects, being suitable for any lab, is characterized by moderate portability and has moderate cost per instrument [25,32,48].

PromethION is a high-throughput, compact, fully integrated device with a computer included for large-scale projects and for centralized sequencing facilities that may accommodate between two and 24 flow cells simultaneously. The capacity of the PromethION flow cell is 6-fold higher than the MinION flow cell, and it generates Tb of data per run (290 Gb/flow cell). It is used for metagenomic (mNGS) and multiomic characterization, human and large-genome sequencing, including whole-genome sequencing (WGS) [32,48].

### 3.5. Sample Types Relevant to the Intensive Care Unit

Nanopore sequencing can be applied to various clinical specimens that are routinely collected in ICUs. However, the diagnostic performance may vary considerably according to specimen type, as microbial load from different samples, inhibitor factors, and sample contamination may occur.

The most frequently collected and analyzed specimen types in ICUs are: whole blood (in the case of sepsis and bloodstream infection, with difficulties encountered because of a low amount of microbial DNA and high amount of host DNA) [11]; positive blood culture (for rapid pathogen identification and antimicrobial resistance profiling) [4]; bronchoalveolar lavage, endotracheal aspirates, and sputum samples (representing the main respiratory samples used in cases with hospital-acquired and ventilator-associated pneumonia HAP/VAP [49,50] and containing large amounts of host DNA, oral microbiota, and colonizing microorganisms); cerebrospinal fluid [51] in cases with meningitis or encephalitis and low pathogen burden, urine, pleural fluid, peritoneal fluid, pericardial fluid, synovial fluid, and abscess aspirates (usually containing reduced amounts of host DNA) [52].

### 3.6. Host DNA Background and Depletion Strategies

Extraction of pure, high-molecular-weight bacterial DNA for the diagnosis of bloodstream infections by using nanopore sequencing is difficult. This is mainly due to the typically low number of circulating microorganisms in bloodstream infections (1–100 CFU/mL), while host DNA is predominant over pathogen DNA because of leukocytosis and the presence of inhibitory substances in blood samples.

Several studies have investigated the challenge problem caused by the increased quantity of host DNA and the low levels of microbial DNA in clinical samples [43]. In this respect, non-ionic detergents, such as saponin, were used to selectively lyse host cells and reduce host DNA contamination while preserving microbial cells [43,53].

A similar challenge is noticed in respiratory samples, which often contain low microbial biomass and high quantities of host DNA. The increased level of host DNA may be due to the presence of epithelial and inflammatory cells, whose DNA can dominate the extracted nucleic acid pool, thereby leading to the relatively low level of microbial DNA available for sequencing [54].

Currently available host DNA depletion methods can be grouped into pre-extraction approaches (that consist of removing host cells and cell-free host DNA before nucleic acid extraction) and in silico approaches, such as nanopore adaptive sampling (which rejects host reads in real time during sequencing). Pre-extraction methods include lysis of host cells, nuclease digestion, size-based physical separation, and enrichment. The most widely used strategy for blood is represented by selective lysis with nuclease treatment: non-ionic detergents (saponin), while the released host DNA is then digested. Moragues-Solanas et al. reported that increasing saponin from 1% to 3% improved depletion of host DNA by an additional order of magnitude without loss of spiked bacteria and that adding a rapid bacterial-enrichment step (SepsiPURE) increased detectable bacteria more than 10-fold, enabling detection down to 1–5 CFU/mL [43]. Consistently, Ali et al. achieved up to a 38-fold reduction in host DNA for Escherichia coli and Staphylococcus aureus using 4% saponin with salt-activated nucleases and showed that shortening bead-beating from 10 to 6 min improved the recovery of longer DNA fragments, indicating how depletion and extraction directly influence sequencing performance [53].

Size-based separation offers an alternative that avoids chemical lysis of microbial cells. Chen et al. evaluated a zwitterionic-interface filtration device that removed more than 99% of white blood cells while allowing bacteria and viruses to pass; sequencing of the enriched genomic DNA detected the expected pathogen in all culture-positive sepsis samples and raised the microbial signal from 925 to 9351 reads per million without altering the microbial composition [55]. Also, Wang et al. found a filtration-plus-nuclease method to be best balanced, whereas some commercial kits removed the most host DNA but obtained the greatest microbial loss and could significantly diminish Prevotella spp. and Mycoplasma pneumoniae [44]. Finally, nanopore adaptive sampling enriches targets in silico during the run, reaching 3- to 5-fold enrichment in blood [56] but only ~3.1-fold in sputum, where rapid pore loss reduced sequencing yield by an estimated 80% [42]. No single method is therefore universally optimal; the choice should be guided by specimen type and microbial load.

## 4. Clinical Applications of Nanopore Sequencing in the ICU

In the ICU, infections are among the leading causes of mortality, necessitating rapid pathogen identification and accurate diagnosis. Nanopore sequencing allows rapid pathogen detection and genomic characterization in clinical settings, particularly in the ICU, where timely diagnosis is critical for appropriate management of critical patients [57].

Accumulating evidence suggests that nanopore sequencing significantly improves diagnostic performance in ICU units for patients with sepsis, bloodstream infections, severe pneumonia, ventilator-associated pneumonia, viral or fungal infections, and infections occurring in immunocompromised hosts [4,13,58]. Oxford Nanopore Technologies enables the direct, real-time analysis of native DNA and RNA from patient specimens at the single-molecule level.

A summary of representative clinical studies evaluating nanopore sequencing in ICU patients is described in Table 1.

### 4.1. Bloodstream Infections and Sepsis

An emerging important clinical application of nanopore technology is the use in critical care/infectious disease [4].

Despite significant developments in antibiotic therapy, drug-resistant bacteria remain a major cause of mortality [62,63]. Bloodstream infections are a major health challenge, especially due to the increasing prevalence of antimicrobial resistance (AMR) [62]. Accurate, rapid identification of pathogens and resistance determinants is important for guiding appropriate treatment and improving patient outcomes. Despite fast advances in critical care, the diagnosis of infections and pathogen identification remain challenging. The gold standard for pathogen detection continues to be represented by blood cultures, although their sensitivity may be influenced by factors such as the timing of blood collection, blood volume, and culture conditions. The sensitivity of blood cultures is substantially reduced following antimicrobial treatment. Furthermore, this approach is time-consuming, and definitive results often require several days [13].

A recent study by Harris et al. included 201 patients (66 positive blood culture samples, of which 52 were sequenced using nanopore technology) and reported that species-level agreement with conventional identification of causative pathogens was 94.2% (100% in monomicrobial infections) [4]. Nevertheless, the study was performed on a relatively small, single-type sample (positive blood cultures) and requires further confirmation in larger, multicenter cohorts [4]. Moreover, Nielsen et al. consider nanopore sequencing a fast, culture-independent tool for the diagnosis of bloodstream infections, capable of identifying a broad range of pathogens missed by conventional blood cultures [59]. Another study reported fast results by using nanopore-targeted sequencing (NTS) in a cohort of 387 blood samples and concordance between NTS and metagenomics sequencing and correlated genotypic AMR in over 80% of cases [60].

### 4.2. Hospital-Acquired Pneumonia and Ventilator-Associated Pneumonia

Hospital-acquired pneumonia (HAP) and its subtype, ventilator-associated pneumonia (VAP), are among the most prevalent healthcare-associated infections in the intensive care unit (ICU) and remain significant causes of morbidity and mortality [54,61]. Proper management of HAP/VAP relies upon timely pathogen identification. Evaluation of respiratory samples using traditional culture-based methods for pathogen detection is slow and limited by prior exposure to broad-spectrum antimicrobial therapy [61].

Wu et al. combined a saponin-based method to remove the host human genome with nanopore sequencing of endotracheal aspirates and observed that, across seven target pathogens, sensitivity of metagenomic sequencing was approximately 2.4-fold higher than culture (89.2% vs. 37.8%), with 98.8% specificity and a short (about 6 h) total workflow [54].

Thus, the reduced number of target pathogens evaluated limits generalizability to the full spectrum of respiratory pathogens encountered in critical care patients from the ICU.

The proof-of-concept study of Heitz et al. identified additional pathogens in bronchoalveolar fluid of HAP/VAP patients without an etiologic diagnosis by culture by performing metagenomic sequencing utilizing the Oxford Nanopore MinION system [61].

### 4.3. Fungal and Viral Infections in ICU Patients

A subset of adult ICU patients without neutropenia and without recognized risk factors frequently develop fungal infections, which represent a major mortality risk [64].

Metagenomic sequencing, including nanopore technology, performed on 128 samples from 87 patients was 93% sensitive and 81% specific for clinically relevant pathogens compared with routine testing [22].

A recent study performed by Macip found that nanopore sequencing produces full-length 16S rRNA reads (~1500 bp), allowing improved taxonomic assignment and species-level resolution. Despite historically higher error rates (5–15%), nanopore sequencing technology has shown improved performance in identifying dominant bacterial species and offers significant advantages for real-time diagnostic applications [65].

Nanopore sequencing represents a valuable diagnostic tool for critically ill patients with unexplained respiratory failure or severe infections in whom conventional pathogen identification tests have failed. Nanopore sequencing may detect various viral pathogens and simultaneously identify viral co-infections that may influence disease severity and treatment decisions in the ICU [15].

### 4.4. Antimicrobial Stewardship and Resistance Detection

Antimicrobial resistance (AMR) represents one of the most pressing challenges for modern healthcare, particularly in the ICU. Beyond pathogen detection, nanopore sequencing enables rapid characterization of antimicrobial resistance factors, offering significant benefits for antimicrobial stewardship in ICUs.

ICUs are particularly susceptible to the emergence and dissemination of multidrug-resistant organisms due to the extensive use of invasive devices, immunosuppressive therapies, and broad-spectrum antibiotics [66].

Pathogens such as methicillin-resistant Staphylococcus aureus (MRSA), vancomycin-resistant Enterococci, and carbapenem-resistant Enterobacteriaceae remain leading causes of healthcare-associated infection in the ICU, and their detection is frequently delayed when conventional culture-based methods are used.

In a large cross-sectional study of 458 positive blood cultures, nanopore sequencing combined with machine learning-based prediction enables rapid identification of resistant pathogens and proved high concordance with phenotypic antimicrobial susceptibility testing across both Gram-positive and Gram-negative organisms, improving patient outcomes [62].

A recent molecular epidemiological study performed in a tertiary ICU identified 62 carbapenem-resistant Gram-negative strains, predominantly Acinetobacter baumannii and Klebsiella pneumoniae, with ERIC-PCR fingerprinting revealing clonal clusters consistent with nosocomial transmission and confirming a strong association between prolonged ICU stay and mortality [67].

Ongoing challenges include variable correlations between genotype and phenotype for specific pathogens and antibiotic pairs, as well as the interpretation of low-abundance resistance determinants, highlighting the need for cautious clinical interpretation of sequencing results [68].

By directly identifying resistance determinants from patients’ samples within hours, nanopore sequencing could enable targeted therapy, reducing unnecessary broad-spectrum antibiotic treatment use and enhancing antimicrobial stewardship and infection prevention efforts.

## 5. Current and Emerging Diagnostic Technologies in Critical Care Medicine

A comparative analysis of clinical applicability of culture-based methods, PCR, short-read next-generation sequencing (Illumina, San Diego, CA, USA), and nanopore sequencing methods across the most frequent and impactful diagnostics encountered in the ICU is presented in Table 2. Biological sample cultures are useful for conventional microbiological confirmation and antimicrobial susceptibility testing; their performance is limited in culture-negative sepsis, fungal infections, and time-critical decision-making. The PCR method enables rapid targeted detection, but its diagnostics application relies on predefined pathogen panels. In contrast, sequencing-based approaches, particularly metagenomic NGS and nanopore sequencing, offer pathogen detection and detection of antimicrobial resistance genes. Nanopore technology provides real-time single-molecule sequencing and has the potential for point-of-care implementation [13].

### 5.1. Comparison of Sequencing Platforms Relevant for ICU Diagnostics

Several sequencing platforms have been developed over the years and used for diagnostics, each based on distinct technological principles. Some platforms are established for short-read sequencing (Illumina and Ion Torrent-Thermo Fisher Scientific, Waltham, MA, USA), others provide high-accuracy long-read sequencing (PacBio-Pacific Biosciences, Menlo Park, CA, USA and Oxford Nanopore, Oxford, UK), whereas at this moment, only one enables portable, real-time sequencing through electrical signal detection (Oxford Nanopore system). These differences are particularly relevant for intensive care diagnostics, where rapid pathogen identification, antimicrobial resistance detection, and early therapeutic decision-making are essential. The main technical and clinical characteristics of sequencing platforms with potential applicability to diagnostics across different conditions in patients from the ICU are summarized in Table 3.

Second-generation technologies (Illumina, Ion Torrent) allow massively parallel sequencing and rely on amplification-dependent sequencing, delivering high per-base accuracy and throughput but only short reads, and require a completed run before analysis [28]. Third-generation sequencing (PacBio, Oxford Nanopore Technology) or high-throughput sequencing allows single-molecule sequencing and is based on changes in molecular weight or electrical signal of incorporated nucleotide during translocation through a pore [24,28,73]. A detailed comparison is available in Table 3.

### 5.2. Advantages of Nanopore Sequencing

Nanopore sequencing technology offers several advantages over conventional sequencing technologies. The ability to preserve native nucleic acid modifications during sequencing represents one of the key strengths of this technology, allowing direct epigenetic analyses without additional sample processing [57].

Moreover, nanopore sequencing allows real-time data acquisition and high accuracy by detecting characteristic ionic current fluctuations generated as target DNA/RNA molecules pass through nanopores. Another major advantage of the SMS technology is the potential to generate long reads [24].

Furthermore, the technology is also attractive for small- and medium-sized laboratories due to its portability, straightforward workflow, and relatively low consumable costs [74]. In addition, nanopore sequencing provides culture-independent pathogen detection and antimicrobial resistance profiling, thereby accelerating laboratory workflows, reducing turnaround times and increasing the demand for fast and precision diagnostics in the ICU [69].

The MinION flow cell may be reused up to 10-fold, allowing lower costs. Furthermore, it is also attractive due to user-friendly bioinformatics pipelines [75].

### 5.3. Challenges, Limitations, and Implementation

Despite significant progress, nanopore sequencing technology faced several barriers that currently limit routine adoption for diagnostic applications in the ICU. Nanopore sequencing is very sensitive to the GC content of reads. High-GC content reads have lower accuracy.

Delahaye et al. showed that nanopore sequencing of short repeated regions, such as homopolymers, as well as more complex STRs, is still challenging. About 50% of sequencing errors (resulting in deletion errors) are due to homopolymers [76].

Although nanopore sequencing provides advantages for long-read analysis, short-read sequencing platforms continue to offer higher basecalling accuracy and may still be preferable for applications requiring high-resolution detection of small-scale variants. However, short-read technologies are less effective in detecting structural variants, highly repetitive genomic regions, and complex genome assemblies, areas in which long-read approaches, including nanopore technology, prove clear advantages [77].

Other critical barriers that limit the widespread adoption of nanopore technologies for diagnostic applications include challenges associated with reproducible nanopore fabrication, relative shelf life, their limited reuse potential, limited spatiotemporal resolution, pore clogging from nonspecific adsorption, control over nanopore dimensions, stability of the nanopore structure, electrical noise, and nonspecific molecular interactions [77,78].

Ongoing challenges include variable correlations between genotype and phenotype for specific pathogens, antibiotic pairs, and interpretation of low-level signals.

Beyond raw sequencing accuracy, reproducibility at the level of clinically actionable results depends on whether sequencing is done under standardized conditions in a single laboratory or across more independent laboratories. Under controlled intra-laboratory conditions (identical chemistry, bioinformatic pipelines), nanopore sequencing has demonstrated high reproducibility, with mean concordance of 99.99% across replicates and similar results for the identification of resistance and virulence markers [79]. Nevertheless, this high reproducibility was not consistent across independent laboratories [80]. A recent multicenter study involving five independent laboratories reported that nanopore-based bacterial genotyping produced strain-specific typing errors in every participating laboratory, with methylation-related artifacts at specific DNA motifs causing inconsistent results even at high sequencing depth, even when using the same R10.4.1 chemistry [80]. Therefore, this reproducibility gap in nanopore sequencing is driven especially by inter-laboratory variability in protocols, software versions, and bioinformatics pipelines, being crucial for ICU settings, where samples may be processed at different diagnostic centers or referred for confirmatory testing.

Rapid library preparation is faster and simpler than ligation-based preparation and is therefore favored in time-critical bloodstream-infection workflows. Several studies reported [43] that rapid-barcoding kits were used precisely for this reason, obtaining results within a few hours (9–12 h) [43,53]. Ligation-based preparation (such as SQK-LSK114) can generate higher output and greater sequencing depth; De Meulenaere et al. showed it tripled on-target base output relative to the earlier chemistry [56] but requires longer hands-on preparation, so the choice reflects a compromise between turnaround time and the yield/sample rather than a fundamental difference in single-read accuracy, which is now dominated by the shared R10.4.1 chemistry. Basecalling similarly affects accuracy: the reviewed studies rely on high-accuracy or super-accurate models (Guppy or Dorado) that improve consensus accuracy and variant calling but add computational time, with super-accurate basecalling preferably performed after the run and capable of adding up to 1–1.5 days [56]. Therefore, final accuracy depends on flow-cell chemistry, the basecalling model, and the bioinformatics pipeline.

Adaptive sampling was reported to have the potential to replace more time-consuming enrichment protocols and is promising in principle for host DNA depletion without additional laboratory processing. Recently, De Meulenaere showed inconsistent real-world performance. While enrichment factors of 3–5 fold have been proved in blood samples with low pathogen burden [56], a recent study performed by Xu et al. in clinical sputum samples reported limited adaptive benefit, shorter read lengths than expected due to the need of PCR amplification, and substantial pore loss during sequencing runs, without a clear cause established [42]. These observations suggest that adaptive sampling’s practical utility remains highly dependent on sample type and is not yet a reliable, widely used solution for host DNA depletion in specimens from critical care patients in the ICU.

However, technical constraints are not the only obstacles to bedside nanopore sequencing in the ICU. Broader barriers also limit the routine clinical implementation of nanopore sequencing in the ICU, including lack of standardized protocols, validated bioinformatic pipelines, and the need for specialized bioinformatics expertise, which is not available in the ICU [81]. In addition, other barriers are represented by the lack of approved in vitro diagnostic kits and clear regulatory pathways (such as FDA approval or CE-IVD).

### 5.4. Emerging Nanopore Technologies and Complementary Rapid Diagnostic Platforms

Although this review primarily focuses on Oxford Nanopore Technologies (ONT), it is important to acknowledge that nanopore sequencing continues to evolve beyond currently available commercial platforms. ONT presently represents the only clinically mature nanopore sequencing technology with widespread adoption in research and increasing implementation in clinical microbiology laboratories. Its combination of portable instrumentation, real-time sequencing, continuously improving chemistry, and established bioinformatics support has enabled its application in infectious disease diagnostics, including critically ill patients. In contrast, several alternative nanopore-based technologies remain under active development but have not yet reached the level of technical maturity required for routine clinical implementation [82].

Among these emerging approaches, solid-state nanopores have attracted considerable interest because of their superior mechanical stability, long operational lifetime, and potential for large-scale manufacturing. Unlike biological nanopores, solid-state nanopores are fabricated using synthetic materials such as silicon nitride or silicon dioxide, allowing precise control of pore dimensions and potentially greater robustness under varying experimental conditions. Nevertheless, signal-to-noise ratios remain inferior to those achieved with biological nanopores, resulting in lower sequencing accuracy and limiting their current clinical applicability [83].

Graphene nanopores represent another promising technology owing to graphene’s exceptional electrical conductivity and atomic-scale thickness, characteristics that theoretically enable single-nucleotide resolution during DNA translocation. However, practical challenges, including rapid DNA translocation through the nanopore, fabrication reproducibility, electrical noise, and difficulties in achieving consistent pore geometry, continue to hinder their translation into routine sequencing platforms. Consequently, graphene-based nanopores remain largely confined to experimental research [84].

In addition, hybrid nanopore systems, which combine biological sensing elements with synthetic nanopore materials, are being investigated to integrate the high sensitivity of biological nanopores with the durability of solid-state devices. Although these systems have demonstrated encouraging experimental results, further optimization of pore architecture, sequencing chemistry, signal processing, and data analysis is required before clinical implementation becomes feasible [85].

Beyond nanopore technologies, several complementary rapid molecular diagnostic platforms are increasingly used in intensive care medicine; however, most require predefined targets, provide limited information regarding novel or unexpected pathogens, or lack the ability to generate long sequencing reads that facilitate genome assembly and comprehensive antimicrobial resistance analysis. In contrast, ONT enables untargeted sequencing directly from clinical specimens while simultaneously providing pathogen identification, antimicrobial resistance gene detection, and genomic epidemiological information within clinically relevant timeframes [86].

Despite the rapid development of these emerging technologies, none currently combines the portability, real-time data generation, flexibility, and clinical maturity achieved by Oxford Nanopore Technologies. Continued advances in nanopore chemistry, pore engineering, sequencing accuracy, and bioinformatics analysis are expected to further expand the role of nanopore sequencing in precision diagnostics. At present, however, ONT remains the only nanopore sequencing platform with sufficient technological maturity, commercial availability, and published clinical evidence to support its implementation in infectious disease diagnostics and intensive care medicine.

## 6. Future Directions

Considering that all the limitations and challenges will be progressively addressed, several promising developments are likely to open broader applications of nanopore sequencing in the diagnostics of critical care patients in the ICU.

One direction is represented by multiomic and protein-level profiling using nanopore sequencing. Nanopore-based single-molecule sensing (SMS) principles are being continuously improved and are starting to be applied toward label-free detection of proteins, peptides, and other biomolecular complexes, becoming an approach that could eventually allow simultaneous pathogen and host analysis on a single instrument. This continuous improvement of the system may ultimately enable, within a single real-time assay, both pathogen identification and characterization of host response.

Another promising future direction is AI-integrated prediction of antimicrobial susceptibility for nanopore sequencing. Machine learning models trained on data obtained from nanopore sequencing (host genome, pathogen genome, and antimicrobial resistance genes) are being developed to identify resistance genes and directly predict antibiotic susceptibility, potentially shortening the path from sample to actionable susceptibility prediction and personalized treatment, especially in polymicrobial or culture-negative settings.

An additional direction is represented by consensus clinical validation frameworks. Development of reference materials, validation of protocols, and standardization of technologies are challenging but critical for integration of nanopore sequencing technology into routine diagnostics in the ICU.

A further direction concerns bringing point-of-care nanopore sequencing closer to the ICU patient. The continued simplification of protocols (such as library preparation), together with the reduction in instrument size and cost for instruments and consumables, is expected to shorten the time from specimen collection to result reporting, therefore supporting real-time diagnosis and implementation into routine ICU practice.

Although recent improvements in nanopore chemistry and basecalling algorithms have enabled consensus accuracies exceeding 99% under optimized conditions, sequencing performance in routine clinical practice remains highly dependent on sample quality, host DNA depletion, sequencing depth, library preparation, and downstream bioinformatic analysis. Consequently, diagnostic performance may vary substantially among different clinical applications.

Despite encouraging diagnostic performance, several important knowledge gaps remain. Most published studies are single-center investigations with relatively small patient cohorts and heterogeneous methodologies. Standardization of laboratory protocols, bioinformatics pipelines, quality-control metrics, and antimicrobial resistance interpretation is still lacking. Furthermore, robust multicenter prospective studies evaluating clinical utility, patient outcomes, and cost-effectiveness are required before nanopore sequencing can be routinely integrated into intensive care diagnostics.

## 7. Conclusions

Nanopore sequencing technology represents one of the most promising molecular diagnostic tools currently available for intensive care medicine. Its unique capacity for real-time sequencing, long-read generation, portability, and culture-independent pathogen detection distinguishes it from conventional diagnostic approaches and makes it a potential key component of future precision critical care.

Current evidence underlines the value of nanopore technology in the diagnosis and management of critical conditions, including sepsis, bloodstream infections, severe pneumonia, ventilator-associated pneumonia, fungal infections, viral infections, and infections in immunocompromised patients. In addition to rapid pathogen identification, nanopore sequencing enables detection of antimicrobial resistance determinants and supports precision medicine by enabling timely and targeted therapeutic decisions.

Although challenges related to nanopore sequencing accuracy, data analysis, standardization, and clinical implementation remain, ongoing advances in this technology are expected to improve diagnostic capabilities and facilitate broader integration into daily ICU practice.

## Figures and Tables

**Table 1:** Representative clinical studies evaluating nanopore sequencing in ICU patients.

Study Design	Sample Size	Specimen	Main Findings	Limitation	Year	Author (Reference)
Prospective, single center	201 patients (52 sequenced)	+ blood cultures	94.2% species concordance (100% monomicrobial)	Single center	2024	Harris PNA et al. [4]
Prospective observational	40 patients	Plasma (cfDNA)	Confirmed all 11 culture + cases; 11 culture negative pathogens	Small cohort	2025	Nielsen ME et al. [59]
Prospective, single center	387 blood samples	Whole blood	Positivity 69.5% vs. 33.9% (culture); AMR concordance 80.6%	Predefined primer panel; single center	2024	Han D et al. [60]
Prospective observational, single center	114 samples/74 patients	Respiratory samples	Sensitivity 97% bacteria, 89% fungi, 89% viruses; therapy changed in 28%	Single center; needs multicenter validation	2025	Alcolea-Medina A et al. [15]
Prospective proof-of-concept	7 target pathogens	Endotracheal aspirates	Sensitivity 89.2% vs. 37.8% (culture); specificity 98.8%; ~6 h	Restricted 7-pathogen panel	2021	Wu N et al. [54]
Proof-of-concept	Small group	Bronchoalveolar lavage	Additional pathogens in culture-negative HAP/VAP	Proof-of-concept; small N	2023	Heitz M et al. [61]
Comparative diagnostic	128 samples/87 patients	Mixed samples	93% sensitivity, 81% specificity vs. routine	Heterogeneous samples	2024	Charalampous T et al. [22]
Cross-sectional, single center	458 positive blood cultures	Positive blood cultures	High concordance with phenotypic AST (Gram±)	Single center	2024	Liu PY et al. [52]
Retrospective, multicenter (10 ICUs)	144 analyses/132 patients	Mixed (mostly CSF, pleural)	Additional pathogen in 25.8%; therapeutic impact 5.3%	Retrospective; long turnaround	2026	Bay et al. [7]

**Table 2:** Comparative analysis of culture-based methods, PCR, short-read NGS (Illumina), and nanopore sequencing methods in the ICU.

Application	Biological Sample Culture	PCR	Illumina	Oxford Nanopore	References
Sepsis	Moderate	Good	Excellent	Excellent	Elbehiry A et al. [69]. Zi GR et al. [70].
Bloodstream infection	Moderate	Good	Excellent	Excellent	Liu PY et al. [62]. Elbehiry A et al. [69]. Zi GR et al. [70]. Papamentzelopoulou M et al. [71].
Ventilator-associated pneumonia (VAP)	Moderate	Good	Excellent	Excellent	Charalampous T et al. [22]. Macip G et al. [65]. Wu N et al. [54]. Lorenzin G, et al. [72].
Fungal infections	Poor	Moderate	Good	Excellent	Charalampous T et al. [22]. Zi GR et al. [70].
Antimicrobial resistance gene detection	Poor	Moderate	Good	Good	Liu PY et al. [62]. Zi GR et al. [70]. Lorenzin G, et al. [72].
Pathogen detection in culture-negative sepsis	No	Limited	High	Very high	Sawale M et al. [20]. Nielsen ME et al. [59]. Zi GR et al. [70].
Time-critical clinical decision support	Slow (Long time)	Moderate	Moderate	Fast/short time (3.63 h)	Wu N et al. [54]. Elbehiry A et al. [69]. Zi GR et al. [70]. Lorenzin G et al. [72].

**Table 3:** Technical and clinical comparison of sequencing platforms for diagnostics in the ICU.

Characteristics	Oxford Nanopore	PacBio	Illumina	Ion Torrent (Thermo Fisher)	References
Principle	Nanopore-based ionic current detection	Single-molecule real-time sequencing SMRT	Sequencing by synthesis SBS	Semiconductor sequencing/pH-based detection	Cui Y et al. [24]. Dongare DB et al. [28]. Zi GR et al. [70]. Arikan A et al. [73].
Read length	Ultra long reads 10 kb–1 Mb	Long reads 10–25 kb	Short reads (150–300 bp)	Short reads (200–600 bp)	Cui Y et al. [24]. Dongare DB et al. [28]. Papamentzelopoulou M et al. [71].
Key features	Third generation, real-time analysis	Real-time single molecule detection	High-throughput	Fast runs, high-throughput	Cui Y et al. [24]. Papamentzelopoulou M et al. [71].
TAT	<1 d (4–8 h)	1–2 d (24–48 h)	1–2 d (24–48 h)	1 d (8–24 h)	Charalampous T et al. [22]. Dongare DB et al. [28].
Real-time sequencing	Yes	No	Partial	No	Cui Y et al. [24]. Zi GR et al. [70].
Sequencing accuracy (quality score)	Improving 99.0% (Q20)/read *	High (99.9%) (Q40)	High (99.9%) (Q40)	High 98.0–99.0% (Q20–Q30)	Cui Y et al. [24]. Dongare DB et al. [28]. Papamentzelopoulou M et al. [71].
Portable device	Yes	No	No	No	Cui Y et al. [24]. Dongare DB et al. [28]. Zi GR et al. [70].
Antimicrobial resistance gene detection	Excellent (database- and depth-dependent)	Excellent	Good	Good	Liu PY et al. [62]. Papamentzelopoulou M et al. [71].
Metagenomic pathogen detection	Excellent	Good	Excellent	Moderate	Charalampous T et al. [22]. Zi GR et al. [70]. Lorenzin G et al. [72].
Hospital workflow integration	Moderate	Low	Moderate	Moderate	Charalampous T et al. [22].
Point-of-care potential	Yes	No	No	No	Cui Y et al. [24]. Zi GR et al. [70].
Critical care applicability	Very high	Moderate	Moderate	Moderate	Charalampous T et al. [22].
Direct RNA sequencing	Yes	Limited	No	No	Cui Y et al. [24]. Zi GR et al. [70].
AI integration potential	Very high	High	High	Moderate	Cui Y et al. [24]. Zi GR et al. [70].
Cost comparison	Moderate (host-depletion kits add cost, higher per Gb, low instrument costs)	High (moderate per Gb, high instrument cost)	Moderate (low per sample at scale, high instrument costs)	Moderate-high (high instrument costs)	Dongare DB et al. [28]. Arikan A et al. [73].

TAT—turnaround time, d—day, h—hours, * Per-read accuracy; consensus accuracy improves after correction but remains below PacBio or Illumina for genome-level assembly.

## Data Availability

No new data were created or analyzed in this study. Data sharing is not applicable to this article.

## Abbreviations

The following abbreviations are used in this manuscript:

ICU	Intensive Care Unit
NGS	Next-Generation Sequencing
tNGS	Targeted Next-Generation Sequencing
mNGS	Metagenomic Next-Generation Sequencing
WGS	Whole-genome sequencing
TAT	Turnaround Time
AMR	Antimicrobial resistance
SMS	Single-Molecule Sequencing
HAP	Hospital-Acquired Pneumonia
VAP	Ventilator-Associated Pneumonia
BAL	Bronchoalveolar lavage
ETA	Endotracheal aspirate
